# The Valedictory Lecture to the Clinical Course on Ophthalmology and Aural Surgery in Cook County Hospital

**Published:** 1868-04

**Authors:** J. S. Hildreth

**Affiliations:** Surgeon to Hospital


ARTICLE XII.

### THE VALEDICTORY LECTURE TO THE CLINICAL COURSE ON OPHTHALMOLOGY AND AURAL SURGERY IN COOK COUNTY HOSPITAL.

By J. S. HILDRETH, M.D., Surgeon to Hospital.

Gentlemen:—Our thirty-first lecture, and last of the present course, is concluded. Many of you will soon leave for fields of practical effort. To get into practice, is the aim with you all. To those about to make the essay, it has become an all-absorbing and anxious thought. Some remarks, bearing upon this subject, seem pertinent to the waning hour.

Practice is the exercise of the comprehension of a subject. Compreheneion may be defined, as taking hold of in the mind; the power of the understanding. Results will correspond to this faculty, other things being equal. When large, its manifestations are powerful; as in the orator, whose polemic eloquence sways the multitude. If refined, the effect will be subtile and delicate; as with the prima donna, whose chant plays upon the heart of her audience. Let the comprehension be selfish and wicked, actions become ugly and brutal; pirates and scoundrels generally, illustrate this. Now, the understanding, or comprehensive grasp of anything, may be diversely acquired:

1st. By trial; as a mason tempers mortar—a manipulation of chemistry, though he may not know the meaning of the word; or, as a laborer learns the use of the lever, in raising heavy bodies—yet ignorant of its mechanical principle.

2d. Philosophically, or by induction and deduction, as Newton grasped the law sustaining the universe; Columbus discovered America.

3d. Accidentally, without forethought, as gold was found in California; Gasthold Schwartz is said to have stumbled upon gunpowder.

4th. From the minds of others, as shown in the moulding influence of association—a man is known by the company he keeps; in the use of books—they are sacks of food for the understanding.

There are still other methods. But, acquire it as one may, comprehension, or knowledge, is the measure of capacity for usefulness and everything else. Manifold causes and conditions make it exceedingly varied in different persons. Let us examine a few:

Take capability. How many have fiddled catgut for a reputation, with no other result than to fiddle away their time; yet, Ole Bull is celebrated throughout Christendom, for his matchless execution on the violin. One artist will produce a portrait that speaks to you; while another, with the same kind of paints, brushes, and canvas, makes a picture nobody recognizes, unless the name be written beneath. Sometimes this quality is intuitive, as with a celebrated mathematician of this city.

Next, see the effect of education. Take two men of equal capacity and bent for a particular calling. Educate one systematically, and let the other come to it, as best he may. Then note the difference, as shown in the career of certain officers in our late war. Innumerable other striking illustrations of this might be cited, but one amply proves the point. Extraordinary ability, alone, can compensate for a lack of systematic training; and, even then, the individual must needs train himself.

Quality of mind, good or bad, a love of truth, or the reverse, wields a potent influence over the intelligence. Minds are frequently found, so small in goodness, it seems as if they constantly dreaded that some truth should penetrate their littleness. Some are ugly enough to refuse to investigate what they fear to be true; thinking, thereby, to extinguish scientific light. Others are so mean as not to admit what they know to be true, lest it benefit somebody beside themselves. And there are still others, too stingy to either learn or let anybody else, if they can help it, spending their time trying to make believe the world has come to a stand-still; that there is no advance in anything, because they, themselves, are incapable of any. Verily, they have their reward.

Let us never forget, however, to do justice to a large and excellent class, who aim to do right, but are enslaved by others, or thwarted by circumstances beyond their control. Such deserve, and must have, a better fate.

Comprehension also varies in kind. There are those who have peculiar knowledge of designing, modeling, or projecting; and, then, there are those specially fitted to grasp the execution of a design, model, or plan. Constantine the Great could found St. Peter's, at Rome; but it took other minds to work it up. An architect models a ship he cannot build; carpenters, blacksmiths, ropemakers, and numerous other workers, must construct the craft. An engineer projects a railway across a chain of mountains; the manufacturer of gunpowder, smelter of iron, rude bosses, driving ruder gangs, with manifold agencies besides, must carry out the plan, or the parallel bars of iron can never unite States, mingle society, and so hold a nation together.

In manufactures and the arts, we frequently see a certain form of comprehension, called aptitude; shown in turning out glassware, compounding silk and woolen dyes, making porcelain, etc.

Another, and very important form of this faculty, is a characteristic trait of men who lead the world: a quality of mind which seeks out, brings together, arranges, and sets to work the requisite materials and forces for a certain result. All successful persons, in anything, possess more or less of this influence. How it preponderates in the life of Washington, Martin Luther, John Jacob Astor, or Stephenson. Great and important enterprises often search long and hard to find a master, with enough of this quality to bring them out. There were competent hands ready to make submarine cable, men to cast it into the Ocean's depths, nautical skill to guide them with their charge, astute electricians to locate break and find defect, vigilant operators, shrewd capitalists, and all other means for the success of the Atlantic cable. This scattered material and personnel had, however, to wait until Cyrus W. Field came to marshal them together, and tie the knot between the old and new continents. During the late rebellion, what gigantic armies were in the field; what fleets upon the seas. Monitors crowded the rivers; innumerable munitions, which made the world wonder, were brought to bear on every side. The nation quaked, to its very foundation, in the struggle for life and liberty. But years, centuries long, were spent before the man was found who could lead our country through the wilderness. This special tact may be termed the power of coördination, or of adaptation of means to a given end. It is by no means always proportionate to strength of intellect, inventive genius, artistic or scientific ability. On the contrary, men of transcending talent have totally failed to make it felt, for the lack of a certain something to bring themselves before the world. It was said of a gigantic intellect, that, in politics, he put to sea on an ebb tide, and was stranded going out. What a museum of valuable inventions, scientific discoveries, artistic productions, poetry, music, and the like, is lost to the world, because its light is hid and nobody dares take the bushel off.

Let us now make an application of the principle we have been illustrating, to the physician.

The practice of medicine is one thing; to huckster drugs, look owlish, wheedle, drive fast, pull teeth, and slaver everybody in reach with unfledged advice is another thing—though frequently supposed by aspirants for public confidence, to be a good imitation. Probably, attached to no other vocation are there more inconsistent notions, weird fancies, and empty ideas mixed up with unsuitable counsel and mistaken effort.

Some think anatomy the keynote of success, and the fuller you sound it the better practice must be. Its importance is too evident to be questioned. But this department teaches the structure of the house you live in, not how to live. Another says, physiology should be the guide of your efforts. It teaches only the uses of parts. Nobody can live by knowing that windows are made to look out of, chimnies to smoke, and the cookstove's function is to digest, and so on through every part of the structure. A third, suggests pathology for the basis of action. Men and women are not wanting who know the pathology of our present system of paper currency. But where is the doctor to cure it? Mathematics, botany, chemistry, and other branches, have their special advocates in this regard. Mathematics aid to tax and appraise, not augment assimilation. Botany will germinate ideas, and big ones too; yet they must have animal heat to thrive, about which chemistry can tell you.

Thus, we might go on, piling citation upon citation; but enough has been adduced to substantiate our point.

Medicine is a concatenation of sciences and an epitome of arts. To practise it successfully, one must marshal and lead these manifold elements, so as to cast in the depths of uncertainty a telegraphic cable between health and disease, to the end that the ailment of the patient may be known and relief dispatched to the suffering organs. In other words, the better you can yoke up medical science and art and then hitch them on to your team, the more weight you will carry through the world as a doctor; whether you attempt to handle the whole system, or concentrate your energies on a part.

Earnest, honest counsel, often fired with eloquence, is delivered to graduating classes. Sometimes, however, I have heard curious advice given to young men, and some not young, as they sat, diploma in hand, to receive the last lecture instalment. I have particularly noticed the suggestion, variously worded, for the newly plumed doctor, "to get on the right side of the women." As if women had any wrong side. Better let them take heed, lest there be so much wrong side to their professional ability that nobody can tell which the right side is. Believe me, that intuitive sense peculiar to women will esteem you, not as a medicine man, but as a *man* in medicine.

Another occasional injunction to graduates is to avoid quackery. The best of advice, but not always understood. Everybody knows what is commonly meant by quackery; but everybody does not know when and where it occurs. Is it not quackery, when a doctor pretends to understand a case, and knows he does not? Is it not quackery, when he treats a case without counsel, and at the same time knows there is somebody within reach of the patient's means, who understands it better than he does? Is it not quackery, for a doctor to send a prescription to a drugstore run by men of indifferent talent, bad habits—laziness, especially—and where whiskey is dispensed behind the counter, or somewhere else, yes, and in the free lunch at 11 o'clock style? Is it not quackery, when the doctor plays upon a patient's hopes by rote, because he cannot read his or her condition by note? Is it not fearful quackery, to become a denominational bigot in sectarian medicine? I will not continue this strain longer, lest it fill the hour.

We have seen how upon one's comprehension of a subject depends his practice. That this comprehension, or taking hold of in the mind—power of the understanding—may be diversely acquired; how exceedingly varied it is in quantity and quality among individuals; and, also, that it comprises a faculty "knowing no obstacle," by which it is allied to practical effort. All this applies with equal force to the acquisition of medical knowledge. Inasmuch as the latter pertains to the welfare of humanity, its study should be governed by a care, delicacy, and patience commensurate with the magnitude of the subject.

The true practitioner of medicine is a cosmopolitan student. He searches truth everywhere, that he may embrace it; and he also searches for error, that he may tread it down. He is a bookish man, and reads all sorts of books relating to his profession—regular, irregular, and defective—and as many more as he can. Like a searching man, he investigates all sorts of systems of medicine: allopathic, homœopathic, eclectic, hydropathic, electropathic, and nothingopathic, in order that he may develope discriminating talent. He is a man of observation, and allows nothing to escape attention, which may enlarge the understanding. His receptive mind always opens the door to entertain knowledge, come from whom or where it may. Taking care to learn but one thing at a time, it is correctly understood, and will be retained. Knowing the importance of executive ability to make knowledge available to the world, he aims to cultivate business talent, etiquette, economy, and politics; that he may know how to live among men. To these, still higher qualities should be added.

On a snowy night, millions upon millions of the white flakes descend from the heavens, though the darkness comprehends them not. Like birds, weary in flight, they lodge in the trees; palisade and stake are crowned as with opal; crystal tops out even the smoke stack; the farmer's soil is shielded from icy blasts; neither pauper-house nor jail is forgotten; for, with philanthropic zeal, these light-winged messengers strive to send them from the face of nature. How earnestly they cling to the charlatan's window, as if fain to repent him of his wickedness. Sidewalks and streets are paved with more than jasper gems. But dawn scarce comes, ere the chimney's new crest is blackened; morning's breath shakes free the branches; horses, tired at early call, nose off the posts; the unwholesome mist from the quack doctor's office despoils the purity of these harbingers of health; they are trodden down by man and beast. Soon this great fleecy mantle will have passed from before our eyes. Yet, ere long, it will again come, to gladden us anew. Therefrom the graceful willow shall spin its slender blossoms; the trees weave their wedding garments; and earth shall spread her velvet carpet to receive the bridal train. What came tiny and gauze-clad, will turn the miller's wheel, bear great ships on its back, and dredge out the river's bed; it will fill the springs, where the cattle slake their thirst in summer's heat; shower gently down, set the bow in the clouds, and enter into myriad forms beside, in nature's great temple.

Gentlemen, the moments of life are snowflakes of eternity. Silently ever-coming, though comprehended not. Like the dove, they bring the olive-branch of peace; infancy and old age are crowned by their radiance; in their strength youth becomes invigorated, and manhood stately; with lavish hand they sustain us in wintry storms of trial; from their fulness, the poor hope for support, and the outcast for justice. These invisible missionaries ever work for the weal of mankind; unweariedly, they search out the heathen in their blindness—wherever they may be—to enlighten them. Scattered broadcast for all, are the priceless jewels of time.

But these visions unceasingly change: full vigor brings decline; the baby's smile turns to wailing; patients pass away; the olive-branch, green and fruitful, fades and withers; waves of adversity sweep away the sands of life; poverty slopes into penury; the exiled and downtrodden look for equity beyond the grave; reformers are ostracized, and barbarians smite philanthropists; opportunities, pregnant with success, are wasted; the barque of life, though launched amid balmy breezes, may go down in a tempest's billow. Yet, out of the incessant roll of the unfathomable mutations of our being, is the living temple built. Though bitter grief come, hopes fade, plans break, effort upon effort fail, friends prove false, society frown, and everything seem to oppose, there is, somehow, a hidden strength in the moments, the finite cannot trace, which may rear the fairest and noblest structure—a well-spent life.

Aim to comprehend and to so practise, that your living temple shall become the pride of the rich and the hope of the poor.

